# Dynamics of *Bactrocera dorsalis* Resistance to Seven Insecticides in South China

**DOI:** 10.3390/insects15090679

**Published:** 2024-09-08

**Authors:** Xinlian Li, Peizheng Li, Doudou Li, Xinyan Cai, Shiwei Gu, Ling Zeng, Daifeng Cheng, Yongyue Lu

**Affiliations:** Department of Entomology, College of Plant Protection, South China Agricultural University, Guangzhou 510642, China; lxl0407000@163.com (X.L.); lfpz@136.com (P.L.); lddoui@163.com (D.L.); xinyan_cai@126.com (X.C.); lynuer@126.com (S.G.); zengling@scau.edu.cn (L.Z.); chengdaifeng@scau.edu.cn (D.C.)

**Keywords:** ecotoxicology, fruit and vegetable pest, invasive species, management, monitoring, pesticide, Tephritidae

## Abstract

**Simple Summary:**

*Bactrocera dorsalis* is an invasive fruit fly pest that causes significant damage to vegetables and fruits in Southern China. Insecticides have been used for many years to effectively control *B. dorsalis*, leading to the development of varying degrees of resistance to a wide range of insecticides. In this study, we monitored the resistance of 11 different populations of *B. dorsalis* to seven commonly used insecticides in Southern China from 2010 to 2013 and followed up in 2023. We found that the resistance dynamics to each of the seven insecticides were unique. Antibiotic insecticides showed the most rapid increase in resistance, while organophosphates exhibited a decreasing trend. These findings will aid in the development of more effective resistance management strategies to improve the efficiency and sustainability of agricultural pest management.

**Abstract:**

*Bactrocera dorsalis* is a highly invasive and destructive pest distributed worldwide. Chemical insecticides remain the primary measure for their control; however, this species has already developed resistance to several insecticides. In recent years, there have been several reports of monitoring *B. dorsalis* resistance in China, but continuous monitoring results are lacking and do not even span a decade. In this study, we monitored the dynamics of resistance to seven insecticides among 11 geographically distinct Chinese populations of *B. dorsalis* (2010–2013; follow-up in 2023). The 11 populations were found to adapt rapidly to antibiotic insecticides (spinosad, emamectin benzoate, and avermectin), reaching high levels of insecticide resistance in several areas. Overall, a decreasing trend in resistance to organophosphorus insecticides (chlorpyrifos and trichlorfon) was observed, whereas pyrethroid (beta-cypermethrin and cyhalothrin) resistance trends were observed to both increase and decrease. The monitoring of field resistance among different *B. dorsalis* populations over the duration of this study is important for improving the efficiency and sustainability of agricultural pest management, and the results provide a scientific basis for the development of more effective resistance management strategies.

## 1. Introduction

Invasive pests present a formidable challenge to agriculture worldwide [1]. *Bactrocera dorsalis* is one of the major invasive quarantine pests affecting fruits and vegetables on a global scale [2,3]. This pest is capable of infecting more than 250 host species, including commercial fruits (such as mangoes and citrus), agricultural products, and wild hosts [4,5,6]. It has spread to 75 countries across Asia, Africa, and Oceania [7], adversely impacting the fruit industry in these regions [8,9]. In China, *B. dorsalis* is widely distributed in the southern regions and is gradually invading and damaging the fruit of various fruit trees in the northern region [10]. Its distribution range is continuously expanding, posing a serious threat to the industry. Moreover, the seasonal distribution of *B. dorsalis* varies according to region. For example, in Fujian Province, the peak occurrence period is July to September [11], while in Yunnan Province, the period of high population growth is March to July [12]. Therefore, in the absence of effective control measures, this pest could potentially cause substantial economic losses in orchard productions [13,14].

Although ongoing research and scientific advancements have led to the development of various fruit fly control methods, chemical agents are still most commonly used to control pests [15,16]. Insecticides have been widely applied in agricultural fields to control *B. dorsalis* outbreaks [17]. In addition to the seven insecticides monitored in this study, many others, such as thiamethoxam [18], bromopropylate [19], and imidacloprid [19], are also commonly used to control *B. dorsalis*. Additionally, based on the damage characteristics and life habits of *B. dorsalis*, the control of adult pests in the field primarily relies on contact insecticides. However, this exposure has led to increased insecticide resistance among *B. dorsalis* populations [20,21]. Comparisons of *B. dorsalis* susceptibility in various regions reveal that the pest has rapidly developed resistance to organophosphates (e.g., trichlorfon) [22,23], pyrethroids (beta-cypermethrin and cyhalothrin) [24], and antibiotics (spinosad and emamectin benzoate) [25,26,27]. In Southern China, at least 10 populations of *B. dorsalis* have developed moderate resistance to organophosphorus insecticides (e.g., trichlorfon) over the past two decades [4,20,28,29]. The increased resistance of *B. dorsalis* to insecticides adversely affects their population control [30,31].

To effectively control *B. dorsalis* using insecticides, the dynamic changes in the resistance of this pest among field populations need to be elucidated. Therefore, in this study, we monitored continuous changes in the resistance of *B. dorsalis* to seven commonly used insecticides (authorized for use in China, http://www.chinapesticide.org.cn/, accessed on 1 June 2024) across 11 different populations (initially during 2010–2013 and again a decade later in 2023). Our goal is to provide detailed information on the insecticide resistance status of *B. dorsalis* and provide a theoretical basis for evaluating the feasibility of these insecticides to manage *B. dorsalis* populations. This information will also provide a reference for designing sustainable resistance management strategies.

## 2. Materials and Methods

### 2.1. Insect Maintenance

A susceptible strain (SS) of B. dorsalis was artificially cultured indoors for 74 generations without exposure to any insecticides, and to determine its sensitivity, the toxicity of the insecticides against the strain was measured several times.

**Figure 1:**
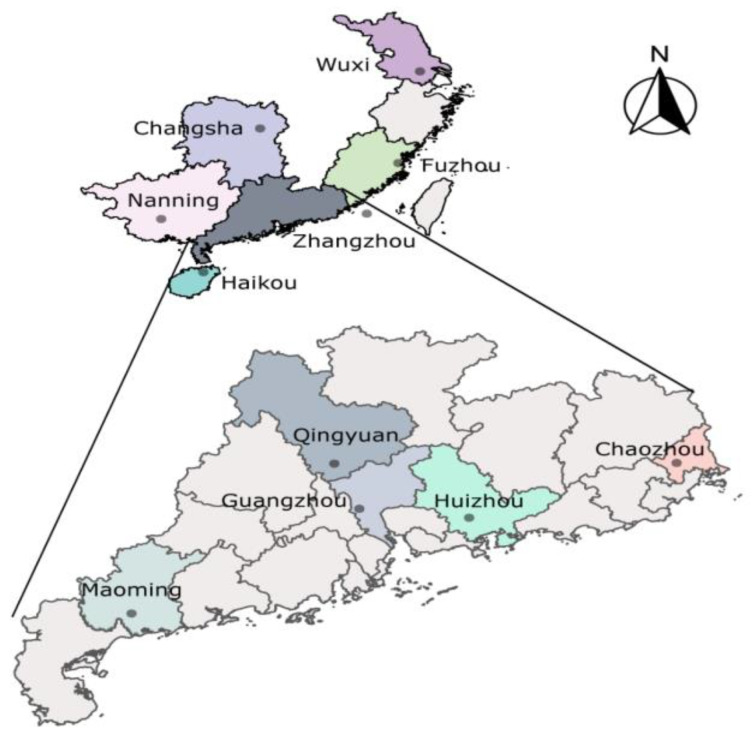
*Bactrocera dorsalis* collection sites.

Field populations (Table 1 and Figure 1) were collected from mango, guava, carambola, and citrus fruits in 11 different regions during July–October 2010, May–October 2011, May–September 2012, and June–November 2013, and a follow-up assessment was conducted a decade later, during July–October 2023. The 3rd instar larvae were removed from collected fruits and placed in soil to pupate. After emergence, the insects were placed inside a rearing cage, where they were provided with an adult diet and water. At this point, all non-*B. dorsalis* individuals were removed to exclude other fruit fly species. *B. dorsalis* eggs were collected using oviposition cups containing orange juice and then placed on the surface of the artificial rearing medium so that the larvae could start feeding directly after hatching. Healthy adults of the F1 generation 3–5 days after emergence were used to determine insecticide toxicity. The populations (SS and field) were reared under the following laboratory conditions: temperature 25–28 °C, light–dark photoperiod 14:10 h, and relative humidity 60–70%. The larval-rearing medium was a mixture of 50 g corn flour, 50 g banana, 0.2 g sodium benzoate, 10 g yeast, 10 g sucrose, 10 g toilet paper, 0.4 mL hydrochloric acid, and 100 mL water. Adults were fed yeast powder and sugar at a ratio of 1:1.

### 2.2. Insecticides

Seven insecticides widely used in Southern China to control *B. dorsalis* populations were selected for bioassay: spinosad, emamectin benzoate, avermectin, and cyhalothrin were purchased from Zhongxun Chemical Co., Huizhou, China. Spinosad (CAS: 168316-95-8, purity ≥ 99.6%); an antibiotic mixture of gastric and thixotropic spinosyns A and D, targets a unique site in the nicotinic acetylcholine receptors of insects; emamectin benzoate (CAS: 155569-91-8, purity ≥ 97.6%); and avermectin (CAS: 71751-41-2, purity ≥ 98.4%), which are also antibiotic insecticides with gastric and thixotropic properties, act on γ-aminobutyric acid (GABA). Cyhalothrin (CAS: 68359-37-5, purity ≥ 97.7%) and beta-cypermethrin (CAS: 86753-92-6, purity ≥ 99.5%, Liwei Chemical Co., Guangdong, China) are pyrethroid insecticides, both of which are contact and stomach toxicants and mainly act on sodium ion channels to kill insects; trichlorfon (CAS: 52-68-6, purity ≥ 99.6%, Nantong Jiangshan Pesticide Co., Jiangsu, China) and chlorpyrifos (CAS: 2921-88-2, purity ≥ 99.9%). Shenzhen RuiDeFeng Pesticide Co., Ltd., Guangdong, China) are organophosphate insecticides with contact and stomach poisoning effects and a main target of action for acetylcholinesterase. The original insecticides were diluted with acetone (CAS: 67-64-1) to obtain master solutions of 100 mg/mL. Each of the freshly configured master solutions was then further diluted with acetone to obtain a series of test solutions at 5–6 different concentration gradients.

### 2.3. Determination of Toxicity

For each concentration of tested insecticides, the following was conducted: after aspirating 5 mL of diluted insecticide solution into a 250 mL conical flask, a uniform film of the insecticide was formed on the wall by rotating the flask; the excess solution was discarded, and then the flask was inverted to allow for the complete evaporation of the acetone. Thereafter, 20 test flies (1:1 sex ratio) were introduced to the flask; an acetone-coated flask was used as the control. Each test used three replicates per treatment. The mortality of flies was checked after 24 h. Flies were considered dead when no activity was observed within 30 s of touching their body. The trial was considered valid when the control group had a mortality rate of less than 10%, and the adjusted mortality was corrected using Abbott’s formula [32]. If the mortality rate of the control group exceeded 10%, the experiment was considered invalid and was repeated.

### 2.4. Statistical Analysis

The experimental data were analyzed using SAS (version 9.4) to calculate the LC_50_ (the concentration at which 50% of *B. dorsalis* were killed), 95% confidence interval (95% CI), and correlation index (R). Resistance ratios (RRs) were calculated as the LC_50_ of the field population divided by that of the SS. Resistance levels were categorized according to the method outlined by Pan et al. (2005) as susceptible (RR < 5.0), low (5.1 < RR < 10.0), moderate (10.1 < RR < 40.0), high (40.1 < RR < 160.0), and extremely high resistance (RR > 160.1).

## 3. Results

### 3.1. Toxicity of Insecticides against the SS

The LC_50_s of the seven insecticides tested against the SS were as follows: 2.52, 1.60, 1.35, 1.35, 0.60, 0.91, and 0.61 mg/L for beta-cypermethrin, trichlorfon, cyhalothrin, emamectin benzoate, spinosad, chlorpyrifos, and avermectin, respectively (Table 2, Table 3, Table 4, Table 5, Table 6, Table 7 and Table 8).

### 3.2. Dynamics of Resistance to Beta-Cypermethrin among Different Populations

Table 2 depicts the resistance levels of different populations to beta-cypermethrin. During 2011–2013, all 11 *B. dorsalis* populations showed moderate resistance to beta-cypermethrin. Resistance of the population from area MM rose from medium to high in 10 years (LC_50_: 57.44 mg·L^−1^, RR: 42.55). Populations from GZ, HZ, QY, NN, HK, FZ, ZZ, CS, and WX had reduced resistance ratios (RRs) in 2023, and some were found to be at the lowest levels during several monitoring exercises (GZ, QY, HK, FZ, and ZZ). Resistance ratios were in the teens in several areas (GZ, QY, HK, FZ, ZZ, and CS), with FZ being the lowest (RR: 10.22), approaching the low resistance level.

### 3.3. Dynamics of Resistance to Cyhalothrin among Different Populations

Resistance to cyhalothrin varied widely among the 11 populations (Table 3). QY and FZ populations were susceptible to cyhalothrin in 2023. Of these, QY previously showed medium resistance from 2010 to 2013, and resistance in FZ previously increased from low in 2010 to medium during 2011–2013. HZ initially showed a large change, from moderate (2010–2012) to high resistance (2013, RR: 42.29), but in 2023 resistance was low (RR: 5.49), almost at the susceptible level. Both NN and WX displayed high resistance in 2023, with RRs of 43.01 and 55.62, respectively. The populations in these two areas showed moderate resistance during 2010–2013. Populations in the remaining six areas (GZ, CZ, MM, HK, ZZ, and CS) maintained moderate resistance throughout the study period. However, the relatively detailed trends show that resistance levels are decreasing in GZ, CZ, and CS while increasing in MM, HK, and ZZ.

### 3.4. Dynamics of Resistance to Trichlorfon among Different Populations

The QY, FZ, and CS populations were all susceptible to trichlorfon in 2023 but had previously been moderately resistant during 2010–2013 (Table 4). The GZ and HZ populations showed low resistance in 2023, declining from medium resistance during 2010–2013. Moderate levels of resistance were recorded in the remaining six districts (CZ, MM, NN, HK, ZZ, and WX) during all five monitoring periods. During 2023, the highest RRs were found in CZ (29.12), NN (25.95), and HK (27.38), and the lowest in MM (10.41), ZZ (12.26), and WX (12.22).

### 3.5. Dynamics of Resistance to Spinosad among Different Populations

The findings for spinosad resistance are presented in Table 5. The FZ population showed an increasing trend in resistance from low in 2010 to medium in 2013, while ZZ and WX maintained low resistance during 2010–2013. By 2023, FZ, ZZ, and WX populations all showed high resistance to spinosad, with RRs of 86.47, 68.23, and 93.32, respectively. The remaining eight areas all showed increased resistance (from low to moderate) across the five monitoring sessions. The slowest increase in resistance was in CS; however, this was the exception. In contrast, other populations showed increased resistance to spinosad, and in seven areas (GZ, HZ, QY, CZ, MM, NN, and HK), the RRs peaked in 2023.

### 3.6. Dynamics of Resistance to Emamectin Benzoate among Different Populations

Table 6 shows that resistance to emamectin benzoate decreased in GZ and QY populations between 2013 and 2023 but was elevated in the rest of the regions, and the highest overall measurements occurred in 2023. The QY population initially showed a decline in resistance from low (2010–2013) to susceptible (2023), and GZ showed little change (2010–2013 and 2023). However, areas ZZ and WX rapidly developed high resistance to emamectin benzoate by 2023 (RRs of 75.20 and 103.79, respectively). In all seven remaining districts (HZ, CZ, MM, NN, HK, FZ, and CS), resistance increased considerably and peaked in 2023 compared to during 2010–2013.

### 3.7. Dynamics of Resistance to Chlorpyrifos among Different Populations

Table 7 shows that 10 of the 11 populations remained susceptible to chlorpyrifos in 2023, except for CZ, which showed low resistance (RR: 5.15) just slightly above the susceptible level (RR of 5). The GZ, FZ, and CS populations were susceptible in all monitoring sessions, and seven populations (HZ, QY, NN, MM, HK, ZZ, and WX) only reached low levels of resistance in one measurement. Overall, resistance to chlorpyrifos remained relatively stable across the 11 populations.

### 3.8. Dynamics of Resistance to Avermectin among Different Populations

Avermectin resistance grew very rapidly in all 11 populations over the decade (Table 8). Even GZ, which exhibited the least growth, doubled in RR during 2013–2023. In the HZ and NN populations, resistance to avermectin increased directly from low to high over the 10 years, increasing 11.12-fold in HZ and by 23.23-fold (the greatest increase) in NN. Except for GZ, all populations showed high resistance to avermectin, with RRs in the CZ and NN groups even exceeding 120.

## 4. Discussion

*Bactrocera dorsalis* can spread over long distances within fruits and vegetables [33], and recent elevations in global temperature have created favorable conditions for its spread, establishment, and outbreaks [34]. Intensive insecticide use in vegetable gardens and orchards is required to effectively control *B. dorsalis* and reduce the economic losses incurred by its infestation during commercial fruit and vegetable production [35]. This has inevitably driven the emergence of resistance in *B. dorsalis*, which explains the ineffectiveness of control using multiple insecticides [36,37,38,39,40,41]. In this study, we monitored changes in resistance to seven pesticides among 11 different Chinese *B. dorsalis* populations between 2010 and 2013 and again a decade later in 2023. Our monitoring results indicate that the resistance types of *B. dorsalis* differed among the 11 field populations.

Monitoring of field populations of *B. dorsalis* in China began in 2003, when the species was sensitive to various agents in most areas [4]. Subsequently, multiple reports indicated that it developed varying levels of resistance to several insecticides. For instance, populations in Shenzhen and Guangxi developed moderate resistance to avermectin (2015) [42,43], while different populations of *B. dorsalis* in Shandong province (Northern China) showed varying levels of resistance to these three types of antibiotic insecticides [44]. In other countries, such as Hawaii, *B. dorsalis* populations showed a widespread, moderate level of resistance to spinosad [45]. In this study, antibiotic insecticides (spinosad, emamectin benzoate, and avermectin) were observed to be highly effective in controlling *B. dorsalis* populations during 2010–2013, with low resistance observed. However, after 10 years, the resistance of *B. dorsalis* to these insecticides has generally increased, showing relatively high levels of resistance in certain areas. This may be due to differences in climate among regions, as well as variations in the number of generations of *B. dorsalis* per year and the types and frequency of insecticide use [46]. Intensive and excessive use of insecticides, inconsistencies in the methods used, and the reference strains employed can all contribute to differences in results [47]. Additionally, the migration of *B. dorsalis* adults with exceptionally high levels of resistance to the sample area in recent years could also have contributed to the increased development of resistance [48]. Therefore, rotating insecticides with different modes of action in the field can reduce the likelihood of resistance development.

For the pyrethroid insecticides beta-cypermethrin and cyhalothrin, moderate resistance was detected in Shenzhen in 2015 [42]; populations in Guangzhou (2005) and Nanning (2018) developed moderate resistance to beta-cypermethrin [28,43].Additionally, *B. dorsalis*, many insects also exhibit resistance to these pesticides, such as *Helicoverpa armigera* [49], *Haematobia* irritans [50], and *Musca domestica* [51]. In this study, resistance levels to beta-cypermethrin and cyhalothrin were both elevated and reduced in different populations. From 2010 to 2023, the resistance of *B. dorsalis* to the two insecticides changed slightly and remained generally stable; however, high levels of resistance were observed in the MM and NN regions. This finding suggests that in South China, beta-cypermethrin and cyhalothrin are still relatively effective chemical insecticides for controlling *B. dorsalis* in Southern China. In regions where high levels of resistance have already or almost been reached, reducing the use of beta-cypermethrin and cyhalothrin or alternating with other insecticides for which *B. dorsalis* have relatively low resistance appears to be acceptable. Therefore, an insecticide resistance management (IRM) strategy needs to be developed to prevent further increase in *B. dorsalis* resistance to these insecticides.

No studies have reported that *B. dorsalis* in China has developed high levels of resistance to organophosphorus insecticides (chlorpyrifos and trichlorfon) locally. Resistance to trichlorfon was mostly moderate, with only a few areas showing low levels of resistance [42,43,52]. For chlorpyrifos, resistance was observed to be mostly low or sensitive [42,43,44]. These results are similar to *B. dorsalis* resistance results for chlorpyrifos and trichlorfon reported in the present study. We found that resistance to the organophosphate insecticide chlorpyrifos tended to stabilize, but an overall declining trend in insecticide resistance was observed. For trichlorfon (another organophosphate insecticide), the CZ, NN, and HK populations showed increased resistance, whereas the remaining eight populations showed increasing sensitivity, similar to the results for chlorpyrifos. These results indicate that chlorpyrifos and trichlorfon are widely suitable for the integrated control of *B. dorsalis* pests in our country.

The evolution of resistance patterns in field pest populations is a complex phenomenon driven by multiple factors [22,53,54,55]. The rapid development of *B. dorsalis* resistance to antibiotic insecticides may be attributed to the low toxicity and rapid action of antibiotic insecticides in mammals, leading to their frequent use as the first choice for pest control [56,57,58,59]. Decreased resistance to organophosphorus insecticides in most populations may be related to integrated management strategies such as bait spraying and killing. Furthermore, rotation with non-cross-resistant insecticides may have helped restore the susceptibility of the populations to insecticides [60,61]. The variability in the resistance of *B. dorsalis* to pyrethroid insecticides may stem from diverse environmental conditions across regions, pest behavior patterns, and varying control strategies employed. However, as seen in this study, the evolutionary patterns of *B. dorsalis* resistance in populations have not been comprehensively elucidated. While our study offers valuable insights into these broader trends, it is important to acknowledge that the absence of specific data on insecticide usage is a limitation of our work. This limitation highlights the necessity for more systematic data collection in future research, which could notably enhance the understanding of resistance dynamics. Moreover, a very wide range of insecticides is used to control the fruit fly, and we only monitored resistance to seven insecticides. Therefore, future studies should focus on expanding monitoring coverage, including investigating additional pest species (such as natural enemies) and insecticide types. There is a need to enhance data integration between field bioassays and laboratory molecular biology tests and to develop and optimize new detection technologies. More scientific and effective pest management strategies should be formulated to ensure pesticide efficacy and environmental safety.

## 5. Conclusions

In this study, *B. dorsalis* field populations were collected from various geographical locations in Southern China, and differences in their levels of resistance to seven insecticides were observed among 11 populations. The study results suggest that in most Southern China regions, the frequent use of antibiotic insecticides, particularly avermectin, should be avoided. Reducing the use of cyhalothrin and beta-cypermethrin or employing alternative insecticides with relatively low resistance is recommended. In Southern China, trichlorfon and chlorpyrifos are effective chemical insecticides for fruitflies. Given the current resistance status observed in fruit flies, appropriate IRM strategies need to be developed for each region. In summary, continuous resistance monitoring, rational rotation of insecticides with other modes of action, and maintaining low resistance levels are crucial strategies for delaying the development of insecticide resistance and controlling *B. dorsalis* populations in a sustainable manner. Additionally, field resistance monitoring of *B. dorsalis* not only contributes to the scientific use of chemicals and pesticide reduction but also promotes integrated pest management while protecting the environment and human health and ensuring the safety of agricultural produce.

## Figures and Tables

**Table 1:** *Bactrocera dorsalis* population collection sites.

Populations	Province	Longitude (E)/Latitude (N)
Guangzhou (GZ)	Guangdong	113°17′/23°8′
Qingyuan (QY)	Guangdong	113°16′/23°49′
Huizhou (HZ)	Guangdong	114°27′/23°10′
Maoming (MM)	Guangdong	110°50′/21°54′
Chaozhou (CZ)	Guangdong	116°38′/23°40′
Nanning (NN)	Guangxi	108°19′/22°49′
Zhangzhou (ZZ)	Fujian	117°59′/23°8′
Fuzhou (FZ)	Fujian	119°28′/26°08′
Wuxi (WX)	Jiangsu	120°29′/31°34′
Changsha (CS)	Hunan	112°58′/28°11′
Haikou (HK)	Hainan	110°19′/20°1′

**Table 2:** Toxicity of beta-cypermethrin against *Bactrocera dorsalis* in Southern China.

Area	2010		2011		2012		2013		2023	
	LC_50_/mg·L^−1^ (95% CI)	RR	LC_50_/mg·L^−1^ (95% CI)	RR	LC_50_/mg·L^−1^ (95% CI)	RR	LC_50_/mg·L^−1^ (95% CI)	RR	LC_50_/mg·L^−1^ (95% CI)	RR
GZ	38.27 (34.88–41.98)	15.21	40.03 (36.61–43.77)	15.91	35.63 (33.96–37.39)	14.14	48.50 (40.15–62.19)	19.25	32.02 (22.21–43.01)	12.71
HZ	70.15 (65.15–75.545)	27.88	83.32 (77.83–89.21)	33.11	65.79 (62.33–69.45)	26.11	75.37 (57.79–120.59)	29.91	57.69 (45.10–79.81)	22.89
QY	53.45 (49.96–57.18)	21.24	48.21 (45.34–51.28)	19.16	58.47 (53.98–33.33)	23.20	47.97 (39.46–62.03)	19.04	33.45 (24.73–44.86)	13.27
CZ	45.85 (42.95–48.94)	18.22	58.03 (54.84–61.41)	23.06	68.53 (64.19–73.16)	27.19	47.69 (38.38–64.38)	18.92	50.63 (43.72–59.47)	20.09
MM	69.05 (61.456–77.58)	27.44	70.78 (64.348–77.86)	28.13	55.61 (51.02–60.61)	22.07	66.81 (57.07–82.54)	26.51	57.44 (49.64–66.95)	42.55
NN	55.86 (53.29–58.55)	22.20	71.26 (66.20–76.70)	28.32	60.79 (55.63–66.43)	24.12	88.94 (79.09–100.01)	35.29	59.37 (50.06–73.14)	23.56
HK	68.31 (63.25–73.77)	27.15	70.19 (65.24–75.52)	27.90	78.04 (73.67–82.68)	30.97	53.59 (42.00–77.31)	21.27	47.56 (37.80–61.39)	18.87
FZ	41.11 (37.84–44.67)	16.34	37.11 (34.31–40.13)	14.75	43.16 (40.22–46.31)	17.13	59.08 (49.12–73.54)	23.44	25.76 (13.31–44.17)	10.22
ZZ	49.49 (45.23–54.15)	19.67	57.65 (52.61–63.18)	22.91	49.44 (46.03–53.10)	19.62	56.55 (43.45–85.83)	22.44	32.73 (21.16–45.91)	12.99
CS	39.02 (36.12–42.16)	15.51	41.62 (38.17–45.39)	16.54	46.15 (42.66–49.91)	18.31	49.09 (39.64–66.00)	19.48	41.45 (35.27–49.32)	16.45
WX	35.44 (32.63–38.49)	14.09	38.34 (35.34–41.59)	15.24	39.78 (37.58–42.12)	15.79	63.35 (52.79–80.26)	25.14	56.68 (47.62–69.84)	22.49
SS	2.52 (2.21–2.88)									

SS: susceptible strain; LC_50_: concentration at which 50% of B. dorsalis were killed; 95% CI: 95% conf; RR: resistance ratios.

**Table 3:** Toxicity of cyhalothrin against *Bactrocera dorsalis* in Southern China.

Area	2010		2011		2012		2013		2023	
	LC_50_/mg·L^−1^ (95% CI)	RR	LC_50_/mg·L^−1^ (95% CI)	RR	LC_50_/mg·L^−1^ (95% CI)	RR	LC_50_/mg·L^−1^ (95% CI)	RR	LC_50_/mg·L^−1^ (95% CI)	RR
GZ	35.84 (33.01–38.91)	26.55	38.57 (35.57–41.83)	28.57	34.39 (32.67–36.19)	25.47	41.74 (34.20–53.22)	30.92	26.60 (21.78–33.78)	19.71
HZ	44.75 (42.19–47.47)	33.15	52.75 (48.42–57.46)	39.07	38.83 (35.95–41.93)	28.76	57.09 (46.36–78.02)	42.29	7.41 (6.32–8.89)	5.49
QY	45.50 (42.87–48.29)	33.70	32.01 (29.45–34.79)	23.71	31.55 (29.64–33.59)	23.37	45.55 (36.79–60.32)	33.74	3.34 (1.92–5.09)	2.47
CZ	46.14 (43.24–49.23)	34.18	38.96 (35.38–42.89)	28.86	43.98 (40.64–47.60)	32.58	43.05 (33.92–58.52)	31.89	38.99 (32.31–46.85)	28.88
MM	36.39 (31.72–41.76)	26.96	37.81 (32.7–43.71)	28.00	39.03 (36.63–41.59)	28.91	35.39 (29.03–43.69)	26.21	52.26 (38.88–70.53)	38.71
NN	21.95 (18.50–26.04)	16.26	29.18 (26.20–32.50)	21.61	31.69 (28.69–35.00)	23.47	30.10 (23.74–37.50)	22.30	58.07 (44.47–78.12)	43.01
HK	24.73 (22.30–27.43)	18.32	24.48 (21.84–37.44)	18.13	25.69 (23.36–28.26)	19.03	34.26 (29.27–40.09)	25.38	38.14 (29.29–49.04)	28.25
FZ	12.81 (10.88–15.10)	9.49	14.44 (12.63–16.51)	10.70	27.77 (25.71–30.01)	20.57	20.01 (15.95–28.74)	14.82	5.70 (4.17–7.72)	4.22
ZZ	22.36 (19.90–25.14)	16.56	27.54 (23.89–31.74)	20.40	30.63 (27.95–3.76)	22.69	34.66 (28.27–42.88)	25.67	39.34 (24.67–60.38)	29.14
CS	39.02 (36.12–42.16)	28.90	26.61 (23.65–29.95)	19.71	26.51 (24.61–28.55)	19.64	23.09 (19.55–26.60)	17.10	19.98 (16.92–23.59)	14.80
WX	15.14 (12.91–17.76)	11.21	24.6 (21.99–27.63)	18.22	32.42 (28.99–36.27)	24.02	30.69 (26.60–38.26)	22.73	75.09 (63.14–94.97)	55.62
SS	1.35 (0.97–1.89)									

SS: susceptible strain; LC_50_: concentration at which 50% of *B. dorsalis* were killed; 95% CI: 95% conf; RR: resistance ratios.

**Table 4:** Toxicity of trichlorfon against *Bactrocera dorsalis* in Southern China.

Area	2010		2011		2012		2013		2023	
	LC_50_/mg·L^−1^ (95% CI)	RR	LC_50_/mg·L^−1^ (95% CI)	RR	LC_50_/mg·L^−1^ (95% CI)	RR	LC_50_/mg·L^−1^ (95% CI)	RR	LC_50_/mg·L^−1^ (95% CI)	RR
GZ	35.81 (29.68–34.50)	22.38	34.61 (31.91–37.53)	21.63	26.08 (24.21–28.09)	16.30	24.68 (22.14–28.89)	15.42	15.414 (13.00–17.97)	9.63
HZ	34.02 (31.80–36.40)	21.24	30.82 (28.64–33.17)	19.24	26.51 (24.43–28.78)	16.57	27.91 (25.23–32.56)	17.44	8.81 (7.14–11.92)	5.51
QY	33.44 (31.14–35.90)	20.88	38.84 (35.25–42.79)	24.25	33.43 (30.25–36.94)	20.89	24.34 (21.75–28.67)	15.21	4.82 (4.12–5.74)	3.01
CZ	38.00 (34.30–42.10)	23.72	37.06 (34.07–0.31)	23.14	41.05 (38.24–44.07)	25.66	21.33 (18.94–24.81)	13.33	46.59 (40.78–53.34)	29.12
MM	44.02 (41.07–47.18)	27.48	47.09 (43.63–50.81)	29.40	42.12 (39.28–45.17)	26.33	35.45 (28.90–48.02)	22.16	16.65 (15.91–17.43)	10.41
NN	35.97 (29.56–36.22)	22.48	34.71 (32.01–37.63)	21.67	37.04 (35.08–39.10)	23.15	25.38 (21.96–32.19)	15.86	41.52 (36.95–46.90)	25.95
HK	20.57 (18.22–23.22)	12.84	20.98 (18.68–23.56)	13.10	25.03 (23.25–26.94)	15.64	20.28 (18.69–22.22)	12.68	43.80 (37.27–51.76)	27.38
FZ	23.05 (20.44–25.99)	14.39	32.58 (29.86–35.56)	20.34	30.20 (27.58–33.07)	18.88	28.02 (24.73–33.39)	17.51	3.33 (3.01–3.70)	2.08
ZZ	33.91 (31.35–36.67)	21.17	32.38 (29.86–35.12)	20.24	33.35 (30.19–36.84)	20.84	27.25 (23.92–32.55)	17.03	19.62 (17.18–22.44)	12.26
CS	31.81 (29.55–34.25)	19.86	35.30 (32.97–37.80)	22.04	25.43 (23.67–27.32)	15.89	26.90 (24.64–30.61)	16.81	3.42 (2.90–4.01)	2.14
WX	27.73 (24.63–31.22)	17.33	32.09 (27.79–37.06)	20.06	28.74 (26.37–31.31)	17.96	23.80 (20.97–27.00)	14.88	19.55 (17.12–22.30)	12.22
SS	1.60 (1.37–1.87)									

SS: susceptible strain; LC_50_: concentration at which 50% of *B. dorsalis* were killed; 95% CI: 95% conf; RR: resistance ratios.

**Table 5:** Toxicity of spinosad against *Bactrocera dorsalis* in Southern China.

Area	2010		2011		2012		2013		2023	
	LC_50_/mg·L^−1^ (95% CI)	RR	LC_50_/mg·L^−1^ (95% CI)	RR	LC_50_/mg·L^−1^ (95% CI)	RR	LC_50_/mg·L^−1^ (95% CI)	RR	LC_50_/mg·L^−1^ (95% CI)	RR
GZ	4.18 (3.44–5.07)	6.97	4.87 (3.92–6.06)	8.12	5.55 (4.73–6.52)	9.25	5.21 (3.93–8.14)	8.68	23.00 (18.18–29.00)	38.33
HZ	5.16 (4.12–6.46)	8.60	4.82 (4.00–5.80)	8.03	5.78 (5.29–6.33)	9.63	5.86 (4.88–7.79)	9.77	23.99 (16.84–33.08)	39.98
QY	4.69 (3.80–5.78)	7.82	5.85 (5.05–6.77)	9.75	6.62 (5.99–7.32)	11.03	7.12 (5.59–10.80)	11.87	12.63 (5.44–18.74)	21.05
CZ	4.49 (3.98–5.05)	7.48	4.53 (4.03–5.08)	7.55	5.49 (5.07–5.95)	9.15	7.37 (6.24–9.45)	12.28	11.07 (6.03–16.34)	18.45
MM	4.41 (3.71–5.24)	7.35	4.96 (4.10–6.00)	8.27	7.10 (6.30–8.01)	11.83	6.73 (5.68–8.48)	11.22	14.85 (8.67–21.35)	23.92
NN	3.95 (3.35–4.66)	6.58	5.25 (4.52–6.10)	8.75	6.83 (6.08–7.67)	11.38	4.70 (3.80–6.26)	7.83	17.73 (15.83–20.01)	29.55
HK	5.36 (4.33–6.64)	8.93	4.98 (4.12–6.03)	8.30	6.47 (5.82–7.18)	10.78	6.20 (5.42–7.09)	10.33	16.36 (11.53–22.05)	27.27
FZ	4.12 (3.40–5.00)	6.87	4.96 (4.22–6.87)	8.97	4.86 (4.51–5.25)	8.10	6.18 (4.70–9.74)	10.30	51.88 (44.54–61.74)	86.47
ZZ	5.53 (4.74–6.47)	9.22	4.25 (3.77–4.79)	7.08	5.35 (4.92–5.82)	8.92	4.65 (3.63–6.55)	7.75	40.94 (27.78–60.43)	68.23
CS	3.67 (3.02–4.45)	6.12	4.75 (3.90–5.78)	7.92	4.34 (3.84–4.91)	7.23	6.91 (5.19–11.37)	11.52	6.44 (5.14–8.41)	10.73
WX	4.68 (3.77–5.80)	7.80	5.23 (4.31–6.36)	8.72	5.13 (4.73–5.56)	8.55	5.63 (4.46–8.00)	9.38	55.99 (48.91–65.24)	93.32
SS	0.60 (0.51–0.71)									

SS: >susceptible strain; LC_50_: concentration at which 50% of *B. dorsalis* were killed; 95% CI: 95% conf; RR: resistance ratios.

**Table 6:** Toxicity of emamectin benzoate against *Bactrocera dorsalis* in Southern China.

Area	2010		2011		2012		2013		2023	
	LC_50_/mg·L^−1^ (95% CI)	RR	LC_50_/mg·L^−1^ (95% CI)	RR	LC_50_/mg·L^−1^ (95% CI)	RR	LC_50_/mg·L^−1^ (95% CI)	RR	LC_50_/mg·L^−1^ (95% CI)	RR
GZ	7.47 (6.47–8.62)	8.30	9.94 (8.80–11.24)	10.99	11.33 (10.51–12.22)	12.59	12.41 (9.89–17.73)	13.79	11.78 (5.23–17.051)	13.09
HZ	6.04 (5.25–6.95)	6.71	7.18 (6.25–8.24)	7.98	10.06 (9.04–11.20)	11.18	6.22 (5.04–8.69)	6.91	13.06 (7.65–18.13)	14.51
QY	4.95 (4.41–5.55)	5.50	7.70 (7.09–8.35)	8.56	7.78 (7.34–8.26)	8.64	6.21 (4.71–9.85)	6.90	3.78 (1.66–5.62)	4.20
CZ	5.86 (5.04–6.81)	6.51	6.26 (5.39–7.27)	6.96	7.59 (6.72–8.57)	8.43	8.50 (6.90–12.20)	9.44	22.00 (15.11–33.15)	24.44
MM	5.95 (5.31–6.66)	6.61	6.29 (5.57–7.11)	6.99	10.85 (10.23–11.50)	12.06	5.74 (4.90–6.87)	6.38	24.20 (16.59–39.39)	26.89
NN	5.04 (4.43–5.72)	5.60	5.63 (4.97–6.38)	6.26	6.98 (6.48–7.52)	7.76	6.09 (4.55–9.97)	6.77	28.16 (18.31–38.00)	31.29
HK	5.53 (4.85–6.31)	6.14	5.35 (4.57–6.27)	5.94	7.67 (6.85–8.59)	8.52	6.38 (5.00–8.13)	7.09	14.67 (11.25–20.67)	16.30
FZ	2.23 (1.96–2.54)	2.48	2.33 (1.98–2.75)	2.59	6.31 (5.74–6.92)	7.01	6.35 (4.47–12.54)	7.06	17.82 (9.33–28.17)	19.80
ZZ	2.06 (1.67–2.55)	2.29	2.59 (2.17–3.08)	2.88	7.58 (6.65–8.65)	8.43	2.93 (2.40–3.52)	3.26	67.68 (57.47–83.42)	75.20
CS	3.95 (3.59–4.35)	4.39	3.55 (3.24–3.90)	3.94	6.07 (5.50–6.69)	6.74	4.95 (3.93–6.86)	5.50	12.56 (10.71–14.99)	13.96
WX	4.00 (3.44–4.66)	4.44	7.31 (6.38–8.38)	10.99	8.73 (8.05–9.47)	9.70	11.30 (9.45–13.17)	12.56	93.41 (74.26–131.76)	103.79
SS	0.90 (0.69–1.17)									

SS: susceptible strain; LC_50_: concentration at which 50% of *B. dorsalis* were killed; 95% CI: 95% conf; RR: resistance ratios.

**Table 7:** Toxicity of chlorpyrifos against *Bactrocera dorsalis* in Southern China.

Area	2010		2011		2012		2023	
	LC_50_/mg·L^−1^ (95% CI)	RR	LC_50_/mg·L^−1^ (95% CI)	RR	LC_50_/mg·L^−1^ (95% CI)	RR	LC_50_/mg·L^−1^ (95% CI)	RR
GZ	2.60 (2.32~2.90)	2.86	3.20 (2.76~3.72)	3.52	3.21 (2.90~3.55)	3.53	2.41 (1.86–3.03)	2.65
HZ	3.00 (2.61~3.44)	3.30	4.06 (3.68~4.49)	4.46	6.40 (5.79~7.07)	7.03	1.98 (1.15–2.84)	2.18
QY	4.38 (3.93~4.89)	4.81	6.01 (5.43~6.65)	6.60	4.41 (4.15~4.69)	4.85	2.45 (2.10–2.84)	2.69
CZ	3.71 (3.31~4.16)	4.08	4.86 (4.32~5.48)	5.34	6.35 (5.76~6.99)	6.98	4.69 (3.40–6.50)	5.15
MM	2.94 (2.56~3.36)	3.49	4.07 (3.69~4.48)	4.47	6.65 (5.94~7.45)	7.31	2.52 (2.09–3.05)	2.77
NN	3.18 (2.73~3.71)	3.49	4.28 (3.84~4.76)	4.70	5.43 (5.03~5.86)	5.97	1.71 (1.14–2.40)	1.88
HK	3.10 (2.640~3.54)	3.41	4.74 (4.31~5.22)	5.21	4.19 (3.92~4.48)	4.60	2.62 (1.80–3.57)	2.88
FZ	2.52 (2.24~2.84)	2.77	2.92 (2.56~3.34)	3.21	3.99 (3.75~4.25)	4.38	2.76 (1.79–3.91)	3.03
ZZ	2.58 (2.27~2.92)	2.84	4.52 (4.12~4.97)	4.97	5.13 (4.77~5.53)	5.64	1.77 (1.02–2.46)	1.95
CS	2.17 (1.92~2.45)	2.38	3.04 (2.6~3.52	3.34	2.71 (2.49~2.96)	2.98	1.97 (1.47–2.61)	2.16
WX	2.16 (1.91~2.44)	2.37	4.81 (4.33~5.35)	5.29	3.93 (3.60~4.29)	4.32	1.34 (1.12–1.59)	1.47
SS	0.91 (0.75~1.10)							

SS: susceptible strain; LC_50_: concentration at which 50% of *B. dorsalis* were killed; 95% CI: 95% conf; RR: resistance ratios.

**Table 8:** Toxicity of avermectin against *Bactrocera dorsalis* in Southern China.

Area	2013		2023	
	LC_50_/mg·L^−1^ (95% CI)	RR	LC_50_/mg·L^−1^ (95% CI)	RR
GZ	6.10 (4.49~10.35)	10.04	13.66 (2.20–24.54)	22.39
HZ	5.31 (4.05~8.00)	8.74	59.31 (47.95–78.15)	97.23
QY	6.28 (4.83~9.55)	10.34	47.21 (34.66–67.70)	77.39
CZ	8.87 (7.30~11.80)	14.60	74.71 (65.63–86.95)	122.48
MM	11.81 (9.13~18.91)	19.44	47.48 (39.33–57.18)	77.84
NN	3.28 (2.61~4.12)	5.40	76.51 (63.97–91.11)	125.43
HK	8.50 (6.85~11.65)	13.99	50.05 (43.51–56.79)	82.05
FZ	9.16 (7.35~13.67)	15.08	32.56 (21.96–44.70)	53.38
ZZ	15.07 (9.85~41.71)	24.81	56.32 (45.26–74.29)	92.33
CS	13.62 (10.16~22.78)	22.42	52.19 (40.82–67.45)	85.56
WX	10.25 (7.82~16.60)	16.88	55.45 (43.93–75.62)	90.90
SS	0.61 (0.47–0.92)			

SS: susceptible strain; LC_50_: concentration at which 50% of *B. dorsalis* were killed; 95% CI: 95% conf; RR: resistance ratios.

## Data Availability

The data presented in this study are available on request from the corresponding author.
